# Rewritable acidochromic papers based on oxazolidine for anticounterfeiting and photosensing of polarity and pH of aqueous media

**DOI:** 10.1038/s41598-022-13440-6

**Published:** 2022-06-07

**Authors:** Bahareh Razavi, Hossein Roghani-Mamaqani, Mehdi Salami-Kalajahi

**Affiliations:** 1 Faculty of Polymer Engineering, Sahand University of Technology, P.O. Box 51335-1996, Tabriz, Iran; 2 Institute of Polymeric Materials, Sahand University of Technology, P.O. Box 51335-1996, Tabriz, Iran

**Keywords:** Materials for optics, Applied optics, Materials chemistry, Photochemistry

## Abstract

Oxazolidine is a new category of stimuli-chromic organic compounds with unique characteristics in response to polarity, pH changes, water, light, and metal ions that were well-known as solvatochromism, acidochromism, hydrochromism, photochromism, and ionochromism, respectively. Therefore, oxazolidine derivatives have been developed for their potential applications in chemosensors, anticounterfeiting, and rewritable hydrochromic papers. In this study, various oxazolidine derivatives containing hydroxyl and naphthalene substituted groups were synthesized by using two different indolenine compounds. The synthesized oxazolidine derivatives were used for investigation of solvatochromism in different solvents, and also acidochromism in various pHs by using UV–Vis and fluorescence spectroscopies. In addition, the oxazolidine derivatives were coated on cellulosic papers using a layer-by-layer strategy to develop rewritable acidochromic papers for printing of security tags on cellulosic papers by using acidic and alkaline solutions as water-based inks. Therefore, the developed rewritable acidochromic papers could be used as security papers.

## Introduction

Indication of pH as a highly applicable factor in a wide variety of systems including water to environment living media has been known as an important issue. Regular pH monitoring inside cells for timely prediction of cancers disease can be creating a great revolution in biomedical science^1–5^. Quality of the source of water can also be monitored by timely detection of pH. The pH safety of foodstuffs is another important challenge in food industries, which is solvable by using of novel technologies based on photo-responsive materials. Using non-complex systems like organic chromophores as chemosensors can help for faster detection of pH in different media, and their price is lower than the other photoactive materials, such as polymer dots, quantum dots, carbon dots, and alternative nanoscale materials^6–12^. Intelligent systems with the ability of changing color, volume, or intensity of fluorescence emission as the alarms to monitor pH have been useful. Therefore, the conjugate chemical structure of organic photochromic and fluorescent compounds is one of the best candidates for observation of variation of color or fluorescence emission under excitation with various wavelengths of light^13–18^. In addition, the environment polarity can affect intensity of fluorescence emission or coloration of these organic compounds, resulted from interactions between the polar functional groups with their conjugate structures. Related to this issue, the solvatochromism phenomenon in different solvents changes the colorimetric/fluorometric characteristics of conjugate organic structures by their interactions with different solvents and the corresponding variation of media polarity accompanied by hypsochromic (or blue) shift or bathochromic (or red) shift phenomena^19–25^. As a significant polarity parameter in aqueous solutions, variation of pH results in protonation or deprotonation of functional groups in the conjugated organic structures which act as aprotic or protic solvents, and their interactions are similar to the effect of electron-donating or electron-withdrawing groups.

A wide range of photochromic and fluorescent compounds has been used in the recent years for development of the colorimetric and fluorometric chemosensors by using solvatochromism, acidochromism, and ionochromism mechanisms^3,26–30^. Oxazolidine is a new category of conjugated organic chromophores with highly intense fluorescence emission and different colors depending on the functional substituted groups or chemical structure of the raw materials especially indolenine compounds^25,31–36^. Type of the substituted functional groups (electron withdrawing or electron donating) is the main effective factor to control fluorescence emission and coloration phenomena by formation of various interactions, such as polar-polar or hydrogen bonding, between oxazolidine with solvent molecules. Polarity of the media is the second effective factor on the colorimetric and fluorometric properties of such systems. Therefore, the effective interactions between oxazolidine derivatives and the surrounding environment are the most powerful tool for development of chemosensors for colorimetric and fluorometric detection of polarity and pH of living media.

**Figure 1:**
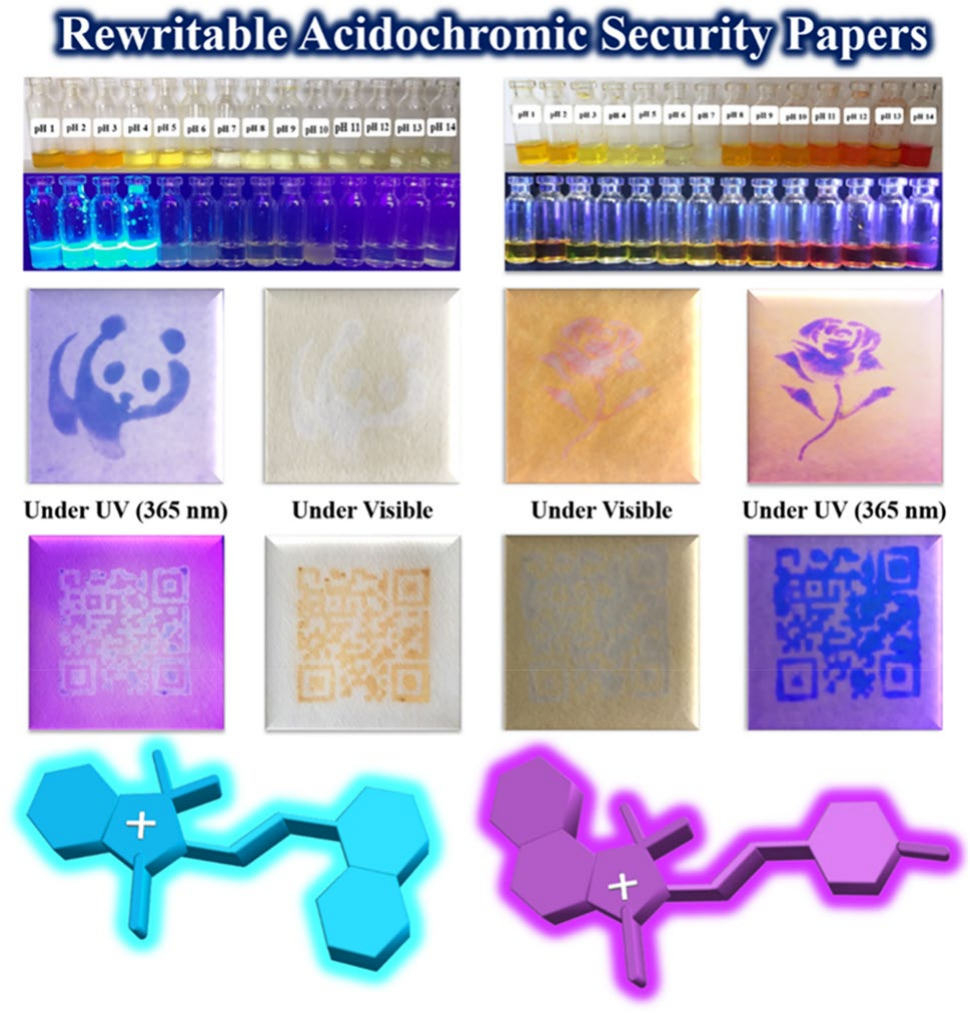
Oxazolidine derivatives for design of rewritable acidochromic security papers printable by acidic and alkaline inks.

In this study, four different types of fluorescent oxazolidine derivatives containing hydroxyl and naphthalene functional groups were synthesized and used for pH monitoring in addition to investigate of their solvatochromism and hydrochromism properties in aqueous solutions. The substituted hydroxyl and naphthalene functional groups on the conjugated oxazolidine have different effects on coloration and fluorescence intensity of chemosensors, which were detected by naked eye without the need for complex and expensive systems. The observed coloration and fluorescence emission can be detected by UV–Vis and fluorescence spectroscopies, respectively. Therefore, colorimetric and fluorometric chemosensors for monitoring of pH and polarity of media were developed based on oxazolidine molecules. In addition, hydrochromism is a well-known unique characteristic for oxazolidine molecules, which can be used for preparation of rewritable anticounterfeiting papers via layer-by-layer coating of oxazolidine solution on cellulosic papers (Fig. 1). Writing on anticounterfeiting papers was carried out by using aqueous solutions with different pHs (acidic or alkaline solutions) as the ink. The printed characters can be erased after evaporation of water molecules, and the writing/erasing cycles are repeated for several times. The oxazolidine solutions can be coated on cellulosic papers to prepare paper-based chemosensors for monitoring pH and polarity of the solvent.

## Experimental

### Materials

2,3,3-Trimethylindolenin (98%), 1,1,2-trimethylbenz[e]indole (98%), 2-bromoethanol (95%), 4-hydroxybenzaldehyde (98%), and 1-naphthaldehyde used for the synthesis of (E)-4-(2-(9,9-dimethyl-2,3-dihydrooxazolo[3,2-a]indol-9a(9H)-yl)vinyl)phenol (OX_1_-OH), (E)-4-(2-(11,11-dimethyl-8,9-dihydrobenzo[e]oxazolo[3,2-a]indol-10a(11H)-yl)vinyl)phenol (OX_2_-OH), (E)-9,9-dimethyl-9a-(2-(naphthalen-1-yl)vinyl)-2,3,9,9a-tetrahydrooxazolo[3,2-a]indole (OX_1_-NaPh), and (E)-11,11-dimethyl-10a-(2-(naphthalen-1-yl)vinyl)-8,9,10a,11- tetrahydrobenzo[e]oxazolo[3,2-a]indole (OX_2_-NaPh) were purchased from Sigma-Aldrich. A wide range of buffers covering pH from 1 to 14 were purchased from Aldrich. All of the solvents including methanol, isopropanol, ethanol, chloroform, tetrahydrofuran, and acetone, supplied from Sigma-Aldrich and Merck Chemical Companies, were used without further purification. Polyethylene glycol (PEG, 3500–4500 g mol^−1^) and NaNO_3_ were purchased from the Merck Chemical Company and used without further purification. Distilled deionized (DI) water was used in all the recipes, and all of the materials were used without further purification.

### Characterization

Chemical structure of OX_1_-OH, OX_2_-OH, OX_1_-NaPh, and OX_2_-NaPh was characterized by proton nuclear magnetic resonance (1H NMR) spectroscopy using a Bruker DPX 400 MHz apparatus in CDCl_3_ solvent. Optical properties of the samples were investigated by UV–Vis analysis by using Jenway 6705 UV/Visible Scanning Spectrophotometer (United Kingdom). Fluorescence emission of the samples was studied by using of JASCO FP-750 Spectrofluorometer (Japan). To evaluate fluorescence properties, the excitation was done by a UV lamp (365 nm, 50 W/m^2^), CAMAG 12VDC/VAC (50/60 Hz, 14 VA, Switzerland). Also, the source for visible light was a common LED lamp (8 W/m^2^).

### Synthesis of oxazolidine derivatives

The oxazolidine derivatives were synthesized according to the procedure reported in the literature^33,35,37^. A solution of 2-bromoethanol (12.5 mmol, 0.9 mL, in 10 mL of 2-buotanone) was added dropwise to the solution of 2,3,3-trimethylindolenin (A, 10 mmol, 1.6 g) or 1,1,2-trimethylbenz[e]indole (B, 10 mmol, 2.1 g) in 20 mL of 2-buotanone solvent and refluxed in N_2_ atmosphere at 80 °C for 48 h to form reddish precipitates, as schematically shown in Fig. 3. The precipitate (C or D) was collected by filter paper and washed three times with acetone to afford reddish solids with a reaction yield of about 60–70%. In the second step, 10 mmol of C (2.85 g) or D (3.35 g) was dissolved in ethanol (20 mL) solvent, and then a solution of 4-hydroxybenzaldehyde (20 mmol, 2.45 g) or 1-naphthaldehyde (20 mmol, 3.12 g) in 10 mL ethanol was added to the mixture and refluxed in N_2_ atmosphere for 48 h. The orange product was filtered after cooling the mixture to room temperature and washing with cold ethanol (5 mL) for three times to obtain E (OX_1_-OH), F (OX_2_-OH), G (OX_1_-NaPh), and H (OX_2_-NaPh) as the oxazolidine derivatives. Chemical structures of oxazolidine derivatives were characterized by FT-IR and 1H NMR analysis (D_2_O and CDCl_3_ as the solvent) and the results are present in the following.

**Figure 2:**
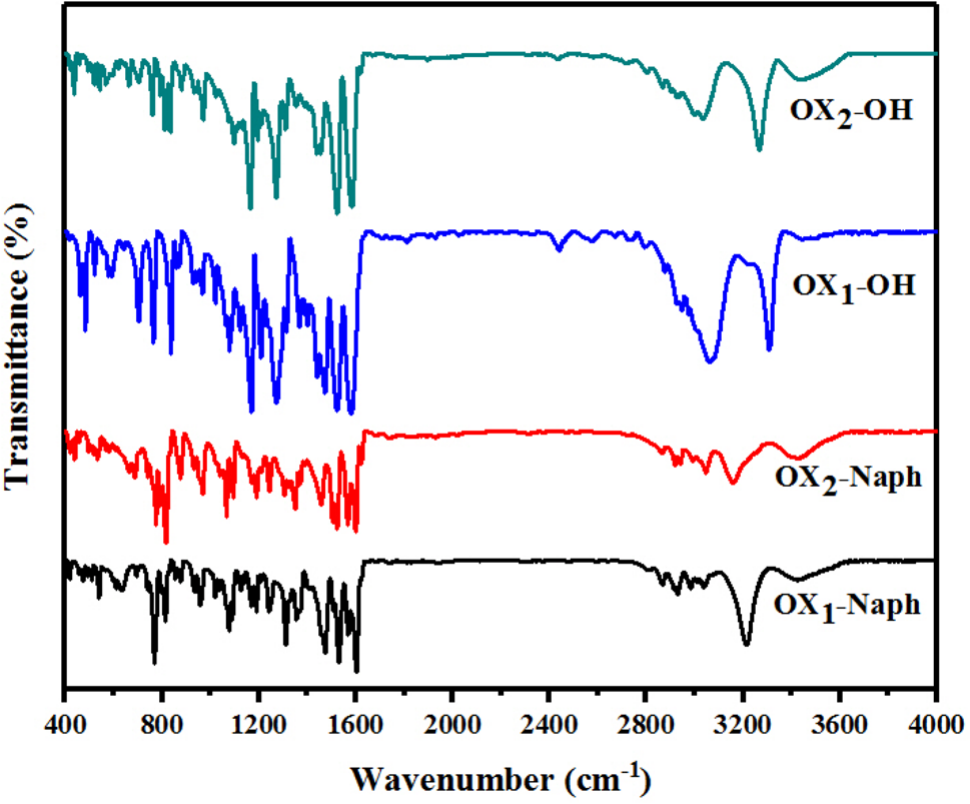
Chemical structure analysis of the oxazolidine derivatives by FTIR analysis.

The main functional groups of oxazolidine derivatives were characterized by FT-IR analysis. According to Fig. 2, the peaks in 3350–3450 cm^−1^ are shown for stretching vibration of aliphatic and aromatic OH groups of OX_1_-OH and OX_2_-OH. Its decreased intensity can be attributed to the proton exchange between the aliphatic hydroxyl groups and aromatic phenolic groups. The aliphatic C–H bond was also observed in 2800–2900 cm^−1^. For the samples OX_1_-Naph and OX_2_-Naph, the sharp band of the aliphatic hydroxyl groups was observed in 3350–3450 cm^−1^. The stretching vibration of aromatic C=C in OX_1_-Naph and OX_2_-Naph was observed in 1500–1600 cm^−1^. The aliphatic C=C band was overlapped with the band of the aromatic C=C, which cannot clearly be observed. The stretching vibration of aromatic C–H units was shown in 3200 cm^−1^.

**OX**_**1**_**-OH**: 

**1H NMR (D**_**2**_**O, 400 MHz, δH (ppm))**: a: 1.69 (1.69 Hz); b: 1.14, 1.17, 1.19 (1.17 Hz); c: 3.06, 3.08, 3.10 (3.09 Hz); d: 3.99, 4.01, 4.02 (4.01 Hz); e: 7.85, 7.86 (7.84 Hz); f: 7.59 (7.54 Hz); g: 7.592 (7.61 Hz); h: 7.495 (7.49 Hz); i: 8.2, 8.25 (8.22 Hz); j: 7.21, 7.22 (7.25 Hz); k: 7.738, 7.766 (7.75 Hz); l: 6.85, 6.88 (6.86 Hz); m: 9.64 (9.60 Hz).

**OX**_**2**_**-OH**: 

**1H NMR (D**_**2**_**O, 400 MHz, δH (ppm))**: a: 1.98 (2.13 Hz); b: 2.13 (4.83 Hz); d: 4.09, 4.08, 4.117 (4.07 Hz); e: 8.387, 8.407 (8.38 Hz); f: 7.59 (7.6 Hz); g: 8.061, 8.092, 8.125 (8.09 Hz); h: 8.312 (8.31 Hz); i: 8.334, 8.32 (7.33 Hz); j: 7.339, 7.395 (7.66 Hz); k: 7.771, 7.795 (7.8 Hz); l: 6.93, 6.954 (6.94 Hz); m: 9.64 (9.64 Hz); n:7.726 (7.75 Hz); o: 7.696 (7.82 Hz).

**OX**_**1**_**-Naph**: 

**1H NMR (CDCl**_**3**_**, 400 MHz, δH (ppm))**: a: 2.01 (2.01 Hz); b: 4.24, 4.29, 4.31 (4.29 Hz); c: 5.07, 5.13, 5.20 (5.13 Hz); p, o, l, f, g, e, j: 7.5- 7.85 (7.82, 7.78, 7.72, 7.68, 7.65, 7.62, and 7.56 Hz); h: 8.02, 8.11(7.96 Hz); k: 8.16 (8.15 Hz); i: 7.92 (8.1 Hz); m: 9.25, 9.30 (9.29 Hz); n: 9.08, 9.14 (9.11 Hz), q: 8.45, 8.50 (8.47 Hz).

**OX**_**2**_**-Naph**: 

**1H NMR (CDCl**_**3**_**, 400 MHz, δH (ppm))**: a:1.62 (1.62 Hz); b: 5.26, 5.27, 5.29 (5.27 Hz); c: 4.33, 4.34, 4.36 (4.34 Hz); d: 4.89 (4.89 Hz); k, l, n, q, p, o, e, f, g, h, s: 7.3- 8.27 (8.29, 8.26, 8.19, 8.15, 7.97, 7.87, 7.81, 7.78, 7.75,7.65, and 7.56 Hz); j: 8.02, 8.11 (6.95 Hz); i: 6.18, 6.20 (6.60 Hz); r: 8.42, 8.45, 8.46 (8.44 Hz); h: 8.66, 8.69 (8.67 Hz), m: 8.72, 8.7 (8.72 Hz).

### Preparation of the ionochromic security papers

The ionochromic security papers were prepared according to the procedure reported by Sheng and coworkers for development of hydrochromic papers by layer-by-layer sterategy^37^. Briefly, high quality filter paper substrate (Whatman qualitative filter paper for technical use, product number: WHA10347512, creped circles with a diameter of 185 mm, Germany) was coated with a thin layer of 10 wt% PEG aqueous solution by impregnation and then drying at room temperature. In the next step, a solution of oxazolidine (0.08 mmol/L) in ethanol/H_2_O (2/3 by volume) containing 6 wt% of PEG and 0.01 wt% of NaNO_3_ was coated on the PEG-coated papers as the second layer. After complete drying of the papers, a layer of 10 wt% PEG solution was coated on these papers as the final layer, and ionochromic security papers were obtained after drying at 50 °C for 4 h. This procedure was used for the preparation of all the ionochromic security papers containing various oxazolidine derivatives.

## Results and discussion

**Figure 3:**
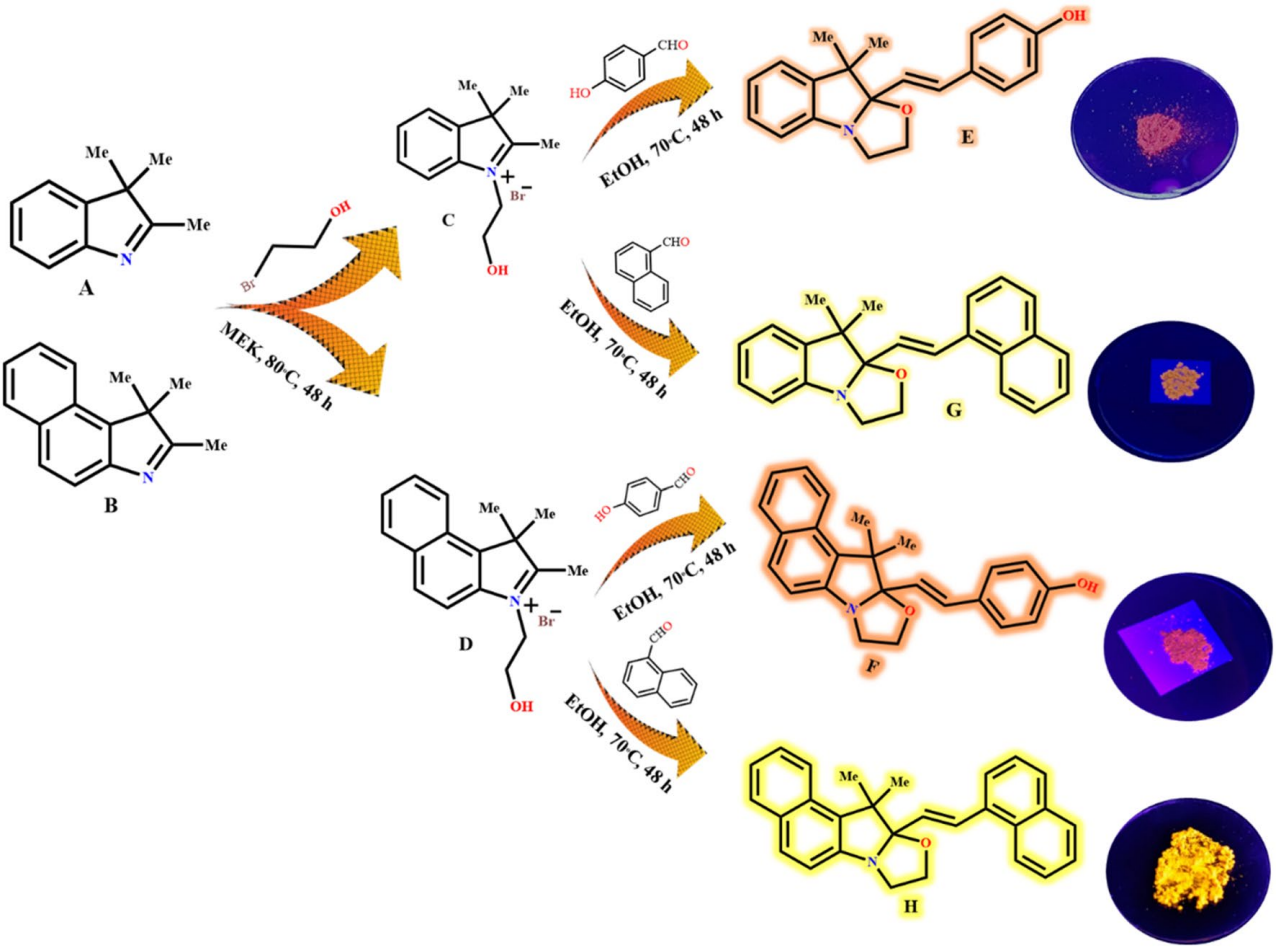
Schematic illustration for synthesis of oxazolidine derivatives from different aldehyde and indolium salts.

Stimuli-chromism phenomena including hydrochromism, solvatochromism, acidochromism and photochromism can be used in design and preparation of chemosensors in solution media. A significant characteristic of oxazolidine derivatives is their water solubility, which resulted in their high importance in development of different chemosensors and also anticounterfeiting materials with high efficiencies in aqueous media^31–33,36–39^. Depending on the substituted functional groups in chemical structure of oxazolidine, a specific fluorescence intensity and color can be obtained. Based on these concepts, four oxazolidine derivatives with different indolenine ring and substituted groups containing hydroxyl and naphthalene functionalities were synthesized, as shown in Fig. 3. Accordingly, intensity and color of fluorescence emission in solid state was higher for F and H, because of higher length of their conjugated structure. According to the spectra obtained from 1H NMR analysis, chemical structure of the oxazolidine derivatives approved their successful synthesis. Effect of different functional groups and their interactions with the media on colorimetric and fluorometric properties in different solvents and pHs are discussed in the following.

### Solvatochromism of the oxazolidine molecules

Solvatochromism as a significant stimuli-chromic phenomenon was observed for the photochromic and fluorescent compounds because of interactions of solute molecules (chromophore or fluorophore) with the solvent molecules. Depending on solvent polarity and its nature, protic or aprotic, a wide range of colors and fluorescence emissions can be observed under visible or UV light irradiation. These solute–solvent interactions are detectable by observation of chromism phenomena, such as bathochromism (red shift), hypsochromism (blue shift), hyperchromism (increasing of intensity), and hypochromism (decreasing of intensity) in UV–Vis and fluorescence spectra. All of the pointed characteristics depend on polarity of solvent and also functional groups of the oxazolidine molecules, consisting of hydroxyl and naphthalene groups in the case of oxazolidine derivatives synthesized in the current work.

**Figure 4:**
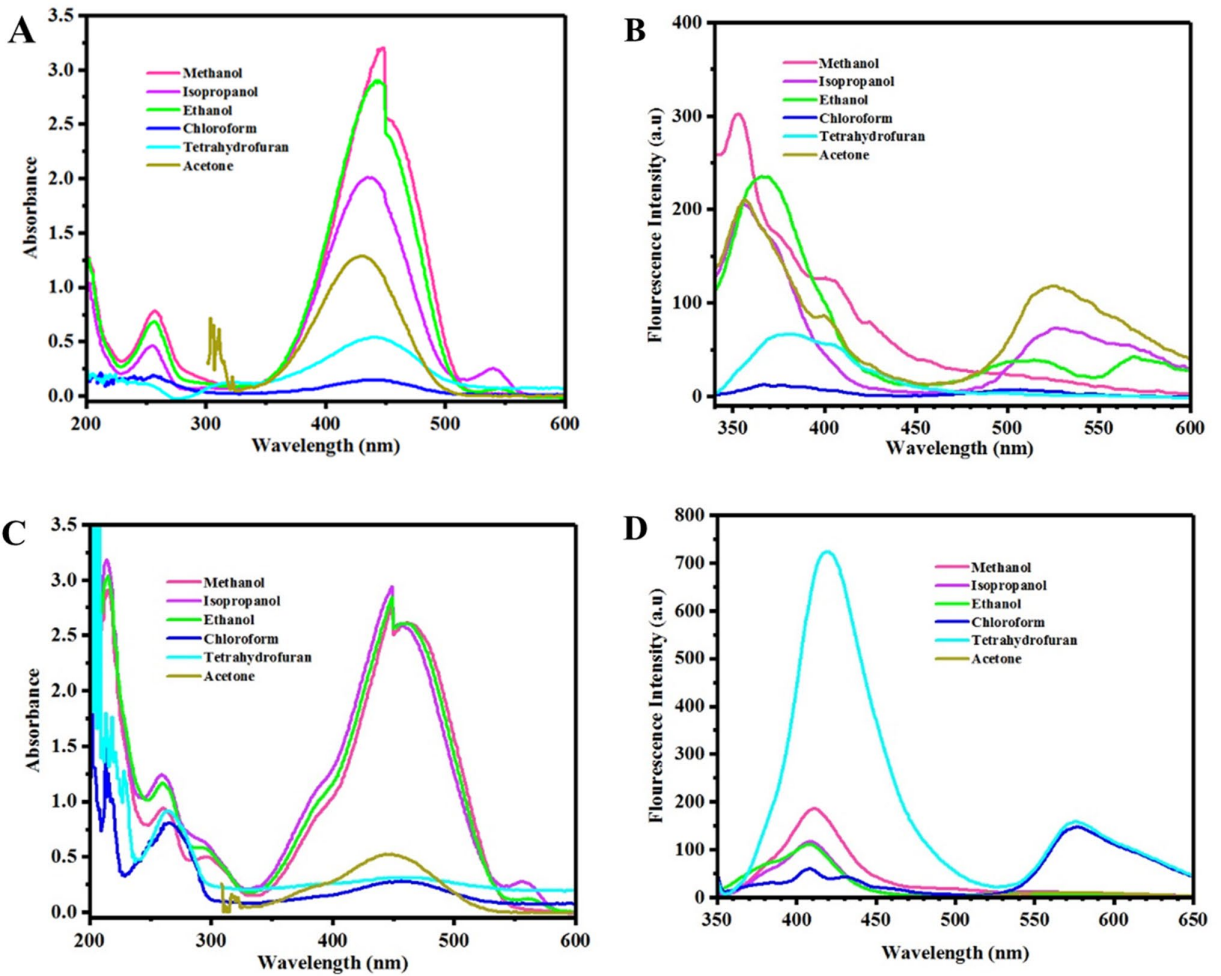
Investigation of solvatochromism behavior in different solvents using UV–Vis and fluorescence spectroscopis for (**A**) and (**B**) OX_1_-OH and (**C**) and (**D**) OX_2_-OH solutions.

Figure 4A displays UV–Vis spectra for OX_1_-OH in various protic and aprotic solvents. Hyperchromism was clearly shown as a function of solvent polarity, where maximum absorbance intensity was observed for the protic solvents including methanol and ethanol with high dielectric constants (33 and 24 at 25 °C, respectively). Chloroform and tetrahydrofuran with low dielectric constants (4.8 and 7.52 at 25 °C, respectively) and low polar indices show the lowest absorbance intensities. The broad absorbance peak in the wavelength range of 300–600 nm is attributed to the color of OX_1_-OH solution in different solvents, where the observed various intensities are due to the interactions of OX_1_-OH chromophores with different solvent molecules by hydrogen bonding (protic solvents) or van der Waals forces (aprotic solvents). These physical interactions act as electron donating and electron withdrawing groups, which affected conjugation and electronic resonance in the structure of oxazolidine derivatives. The pointed phenomena are well-known as solvatochromism that are observable as changing color or its intensity and fluorescent emission or its intensity in the related spectra (UV–Vis and fluorescence) or even by the naked eye. As expected, solvatochromism of OX_1_-OH in protic and aprotic media was led to observe different fluorescence emission spectra in Fig. 3B. Decreasing polarity from protic to aprotic solvents was lad to two emission peaks in fluorescence spectra of Fig. 4B. This complex behavior is resulted from different interactions of OX_1_-OH in different solvents with various polarity and dielectric constants.

The higher conjugation length in chemical structure of OX_2_-OH in comparison with OX_1_-OH significantly affected its optical properties and also stimuli-chromic behavior. As expected, similar trends were observed for the solvatochromism of OX_2_-OH dissolved in protic and aprotic solvents, as shown in UV–Vis spectra presented in Fig. 4C. The OX_2_-OH derivative displays highly-intense fluorescence emission in the solid state that indicated its higher sensitivity to external stimuli, leading to its higher sensitivity to the solvent polarity. Investigation of solvatochromism indicates that OX_2_-OH have maximum absorbance intensity in protic solvents including methanol, ethanol, and isopropanol that resulted from hydrogen bonding interactions between the chromophore and solvent molecules. On the contrary, interactions of OX_2_-OH with aprotic solvent molecules (especially tetrahydrofuran and chloroform) resulted in remarkable hypsochromism by decrease of absorbance intensity in the wavelength of 300–600 nm and also a sharp absorbance peak in 200–300 nm, which can be attributed to higher ability of OX_2_-OH for absorption of UV light than OX_1_-OH. Figure 4D shows fluorescence spectra of OX_2_-OH in different solvents that confirmed the results of UV–Vis spectra. In the case of protic solvents, OX_2_-OH has medium fluorescence emission intensity, where the highest emission was observed for chloroform and tetrahydrofuran. In addition, the OX_2_-OH molecules have a very sharp and intense emission in 350–500 nm and medium emission in 500–650 nm, when dissolved in tetrahydrofuran. This behavior was well-known as two-photon excitation that observed in both of the OX_1_-OH and OX_2_-OH, especially by dissolving in less polar solvents of acetone (OX_1_-OH) and tetrahydrofuran (OX_2_-OH). These results indicate interesting solvatochromism behavior of OX_1_-OH and OX_2_-OH with high sensitivity of solvent polarity, which is resulted from hydroxyl functional groups in their chemical structures that induce highly effective interactions with surrounding media by hydrogen bonding or van der Waals forces.

**Figure 5:**
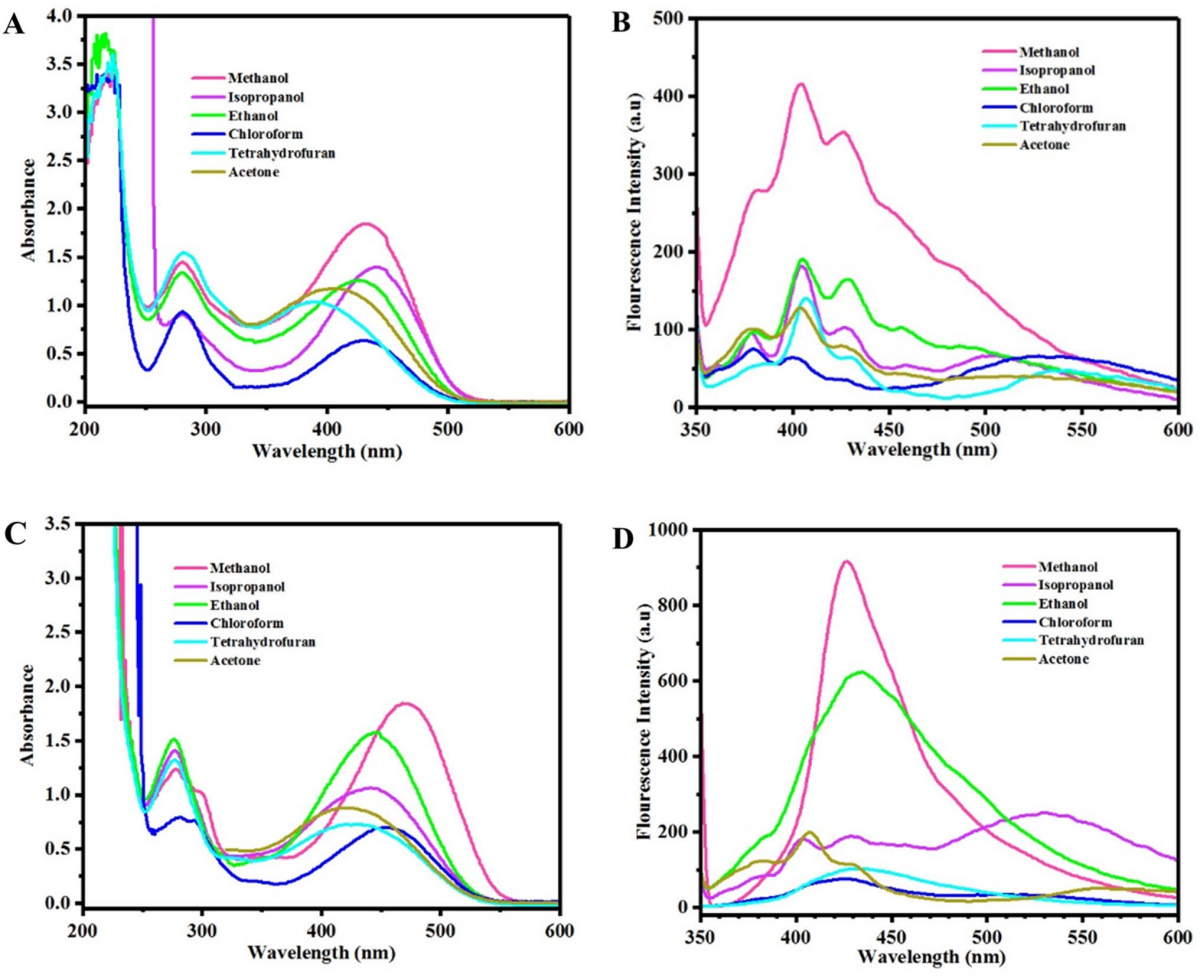
Investigation of solvatochromism behavior in different solvents in UV–Vis and fluorescence spectroscopy for (**A**) and (**B**) OX_1_-Naph and (**C**) and (**D**) OX_2_-Naph solutions.

Figure 5 displays solvatochromism of the OX_1_-Naph and OX_2_-Naph derivatives in both of the protic and aprotic solvents by UV–Vis and fluorescence spectroscopies. Similar to the solvatochromic results obtained for OX_1_-OH and OX_2_-OH, intensity of the absorbance peaks in 250–500 nm for OX_1_-Naph and OX_2_-Naph in Fig. 5A,C, respectively, was increased by increasing the solvent polarity, where the maximum intensity was calculated for the protic solvents especially methanol, ethanol, and isopropanol solutions. The effect of solvent polarity in fluorescence emission of OX_1_-Naph and OX_2_-Naph was also investigated, and the results are presented in Fig. 5B,C, respectively. The fluorescence emission spectra indicate that the emission peaks intensity for OX_1_-Naph and OX_2_-Naph was increased for the protic solvents in comparison with the aprotic ones, where maximum emission intensity was recorded for the methanol solutions of oxazolidine derivatives (Fig. 5B,C). Dependence of the optical properties of OX_1_-OH and OX_2_-OH to the polarity of media was attributed to interactions of phenol hydroxyl groups with solvent molecules, which cannot be connected to solvatochromic behavior of OX_1_-Naph and OX_2_-Naph due to the non-polar nature of naphthalene groups. Therefore, the observed solvatochromism phenomena for OX_1_-Naph and OX_2_-Naph in various solvents can be attributed to the solute–solvent forces, where highly polar solvents have efficient interactions with positively charged nitrogen atom in the indolenine ring of oxazolidine structure. Therefore, the electron-rich solvents such as methanol and ethanol with nonbonding electrons on hydroxyl groups displayed high intensity for fluorescence emission and also maximum absorbance intensity. It is very important that solvatochromic properties of the oxazolidine derivatives can significantly be influenced by substituted functional groups on their chemical structure, the length of conjugation or electron resonance, and also nature of the functional groups (electron withdrawing or electron donating).

### Hydrochromism of the oxazolidine derivatives

**Figure 6:**
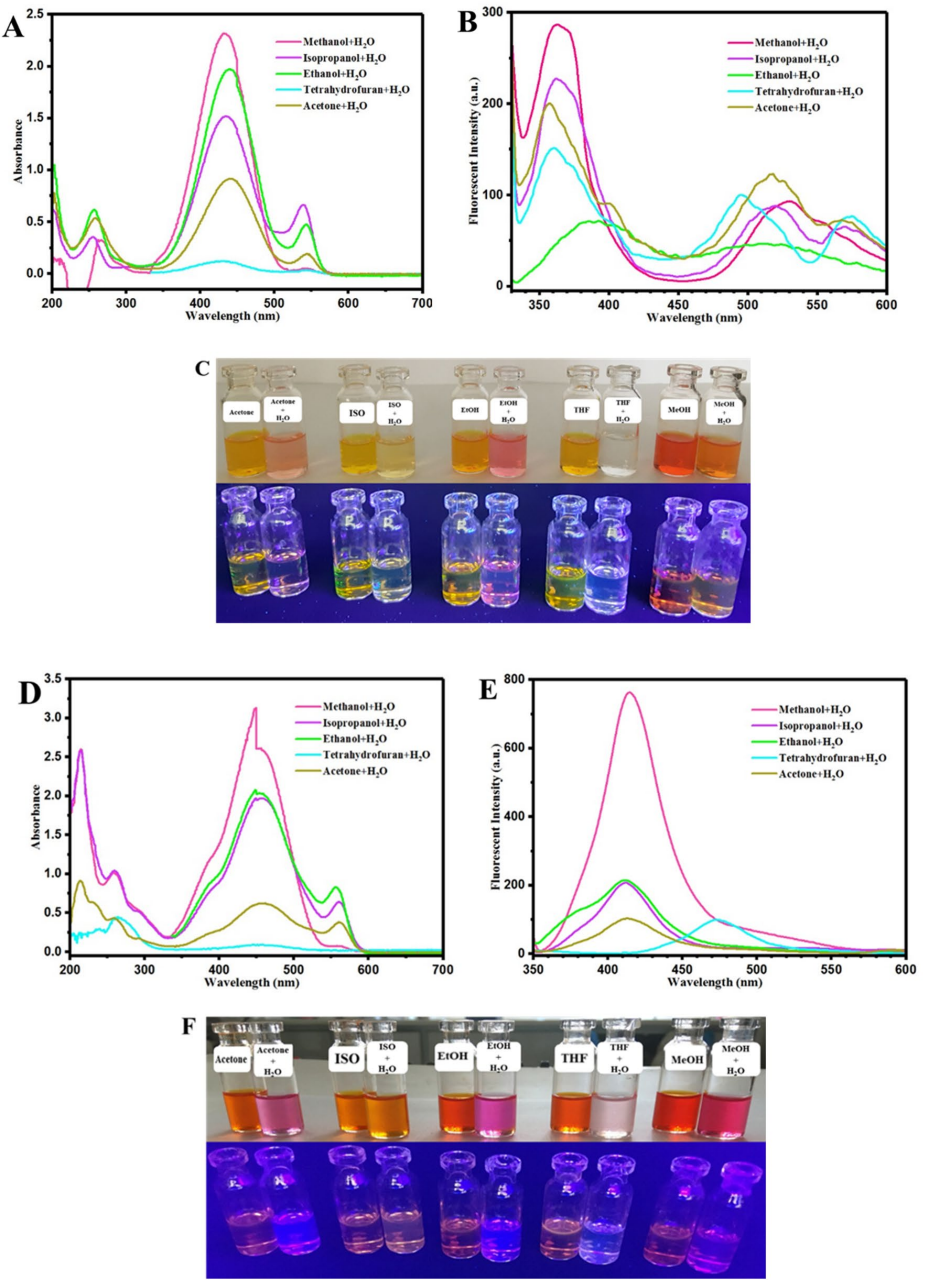
Hydrochromism plots in UV–Vis and fluorescence spectroscopies for (**A**) and (**B**) OX_1_-OH and (**D**) and (**E**) OX_2_-OH solutions and also photographs of hydrochromism phenomenon for (**C**) OX_1_-OH and (**F**) OX_2_-OH solutions before and after addition of water under visible light and UV irradiation (360 nm).

Hydrochromism is a well-known and interesting phenomenon for oxazolidine derivatives that coloration or color change can be observed for dye solutions after addition of water. It is a novel phenomenon that was extensively used for development of rewritable hydrochromic papers, where water was used as the ink for water-jet printing for several times^40,41^. Hydrochromic properties of oxazolidine derivatives were studied by UV–Vis and fluorescence spectroscopies. For this purpose, all of the oxazolidine molecules were dissolved in solvents which are miscible with water, then water was added to the solutions (with a solvent/water ratio of 2/1). The resulted oxazolidine solutions were used for investigation of hydrochromism, and the obtained UV–Vis and fluorescence emission spectra are presented in Fig. 6 in the case of OX_1_-OH and OX_2_-OH derivatives. As discussed in the previous section, protic solvents have significant effect on the optical properties of the oxazolidine derivatives because of hydrogen bonding interactions between the solute and solvent molecules^36,37^. A remarkable change in UV–Vis and fluorescence spectra was observed after the addition of water into the OX_1_-OH and OX_2_-OH solutions, as shown in Fig. 6A,B, respectively. Adding of water to the oxazolidine solutions resulted in a new peak in the absorbance spectrum in the wavelength of 500–600 nm (Fig. 6A) in the case of isopropanol, ethanol, and acetone. On the other hand, similar polarity of methanol and water resulted in insignificant changes in UV–Vis spectrum of methanol/water in comparing with the methanol solution of OX_1_-OH. A significant change in emission spectra was observed for OX_1_-OH in water/solvent media in comparison with the pure solvent media that confirmed the UV–Vis spectroscopy results, and it can be attributed to significant effect of hydrochromism on color change and fluorescence emission of the oxazolidine molecules. Figure 6C shows the photographs of OX_1_-OH solution before and after addition of water under visible and UV light irradiation (365 nm). In agreement with the results of UV–Vis and fluorescence spectroscopies, the OX_1_-OH solutions showed a clear and intense change in color and fluorescence emission after addition of water. Such a hydrochromism is visible by naked eye, and has potential applications in development of humidity or pH chemosensors and also anticounterfeiting technologies.

As shown in Fig. 6D,E,F, OX_2_-OH displayed hydrochromic properties with high intensity in comparison with OX_1_-OH, after addition of water and formation of hydrogen bonding between the solute and solvent molecules. A remarkable decrease in absorbance intensity in the wavelength of 300–500 nm was observed for OX_2_-OH in THF solution after addition of water, which is associated with discoloration of the solution, similar to the results of OX_1_-OH. Investigation of hydrochromism for OX_2_-OH by fluorescence spectroscopy (Fig. 6E) indicated a decrease in fluorescence emission after adding water for all of the samples except than methanol, because of close polarity of methanol and water. Figure 6F showed the photographs of hydrochromism phenomenon for OX_2_-OH under visible light and UV irradiation (360 nm). Similar results were observed for OX_2_-OH compared to OX_1_-OH with high color intensity.

**Figure 7:**
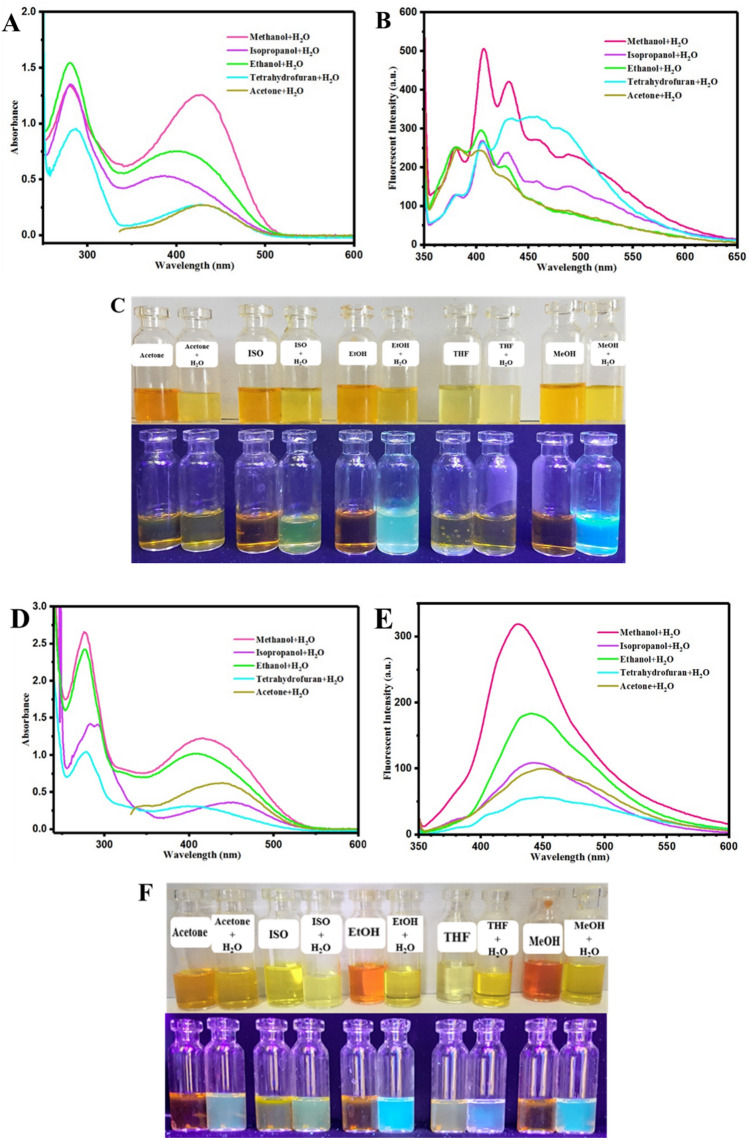
Hydrochromism plots in UV–Vis and fluorescence spectroscopy for (**A**) and (**B**) OX_1_-Naph and (**D**) and (**E**) OX_2_-Naph solutions and also photographs of hydrochromism phenomenon for (**C**) OX_1_-Naph and (**F**) OX_2_-Naph solutions before and after addition of water under visible light and UV irradiation (360 nm).

Investigation of solvatochromism for OX_1_-Naph and OX_2_-Naph displayed lower dependency of optical properties to the solvent polarity in comparison with the OX_1_-OH and OX_2_-OH derivatives, which is resulted from higher sensitivity of the hydroxyl groups to polarity by formation of hydrogen bonding with the solvent molecules. Study of hydrochromism phenomenon for OX_1_-Naph and OX_2_-Naph was carried out by UV–Vis and fluorescence spectroscopies in a similar condition with the previous samples, and the obtained results are presented in Fig. 7. As predicted for OX_1_-Naph and OX_2_-Naph, the UV–Vis spectra in Fig. 7A,D indicted a medium change in absorbance intensity in the wavelength of 350–550 nm after the addition of water, where the most polar solvents (protic solvents) have maximum absorbance intensity. However, a remarkable increase of intensity was recorded for the absorbance peak in 250–350 nm (especially for ethanol, methanol, and isopropanol) in UV irradiation region, and the fluorescence emission of OX_1_-Naph and OX_2_-Naph was significantly affected by hydrochromism. Figure 7B,E showed a clear change in the fluorescence emission spectra of OX_1_-Naph and OX_2_-Naph resulted from hydrochromism and formation of hydrogen bonding between the positively-charged nitrogen atom in indolenine ring and water molecules. The maximum intensity of fluorescence emission changes is related to OX_1_-Naph in methanol/water and ethanol/water solutions, and also for OX_2_-Naph when dissolved in methanol/water and THF/water media. The photographs obtained from investigation of hydrochromism of OX_1_-Naph and OX_2_-Naph in different solvent/water mixtures are presented in Fig. 6C,F. In agreement with the results of UV–Vis and fluorescence spectra, hydrochromism for OX_1_-Naph was led to highly-intense cyan blue fluorescence emission especially in the case of methanol/water and ethanol/water media and also color change/fluorescence emission was changed for all of the samples (Fig. 7C). On the other hand, the samples based on OX_2_-Naph displayed color change and fluorescence emission after addition of water, which a highly-intense and bright cyan blue fluorescence emission was observed for the samples in ethanol/water, methanol/water, and THF/water media. The results of hydrochromism study for all of the samples are useful for development of chemosensors for detection of pH or humidity and most importantly to design novel anticounterfeiting systems based on hydrochromic rewritable papers^42^.

### Photodetection of pH by oxazolidine derivatives

**Figure 8:**
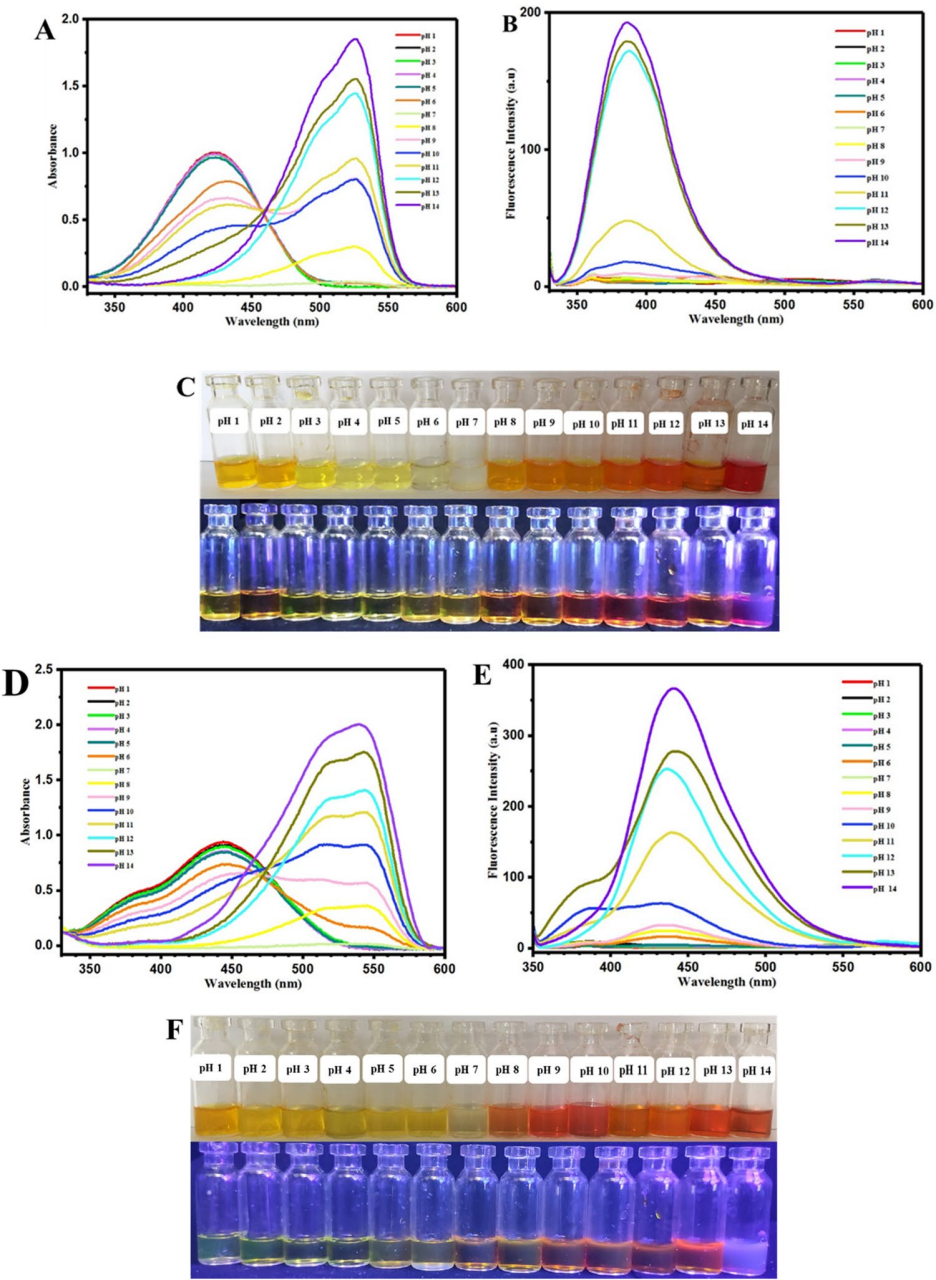
Photodetection of pH using UV–Vis and fluorescence spectroscopies for (**A**) and (**B**) OX_1_-OH and (**D**) and (**E**) OX_2_-OH solutions and also photographic images of colorimetric and fluorometric properties of (**C**) OX_1_-OH and (**F**) OX_2_-OH at pH of 1–14.

Acidochromism of oxazolidine chromophores is another important type of their stimuli-chromic characteristics that was studied for development of anticounterfeiting technologies based on acidic or alkaline solutions of oxazolidine derivatives^16,18,33^. In addition, polarity sensitivity of oxazolidine molecules in aqueous media resulted in their applicability as a chemosensor for colorimetric and fluorometric photosensing of pH. Therefore, oxazolidine solutions in acidic and alkaline media were prepared in the pH range of 1–14 and studied by UV–Vis and fluorescence spectroscopies. As shown in Fig. 8A, the OX_1_-OH aqueous solutions at various pH values have different absorbance spectra, and the broadest and intense absorbance peak was observed for the acidic pHs in 330–500 nm. It was clearly observed that intensity of the absorbance peak in 330–500 nm was decreased by increasing pH to alkaline conditions, and pH 7 displayed minimum absorbance intensity (about 0) for this area. For pH values of higher than 7, a significant bathochromic phenomenon (red shift) was clearly observed that caused to appear a new absorbance peak with higher intensity in 400–600 nm. Its intensity was increased as a function of alkaline strength of media, and the maximum intensity is related to pH 14. Investigation of fluorescence emission of the OX_1_-OH solutions with different pH values was carried out by fluorescence spectroscopy, and the results are presented in Fig. 8B. The OX_1_-OH solutions with acidic and neutral pH (in the range of 1–7) displayed minimum fluorescence emission intensity due to protonation of the hydroxyl groups and decrease of electron resonance in the conjugated structure of OX_1_-OH. On the other hand, increasing of pH to alkaline conditions (in the range of 8–14) was led to increase of fluorescence emission intensity especially for pH in the range of 10–14, and maximum emission was calculated for the pH of 12, 13, and 14. This phenomenon can be attributed to the deprotonation of phenolate hydroxyl groups in alkaline solutions, which induced higher density of negative charges to OX_1_-OH structure and correspondingly increase of electron resonance in its conjugated structure^31,32,38,43^.The photography of colorimetric and fluorometric properties of OX_1_-OH at pH of 1–14 are presented in Fig. 8C. The observations indicated different colors, fluorescence emissions, and also intensities for the OX_1_-OH solutions, which have potential applications for colorimetric and fluorometric photosensing of pH in aqueous solutions.

Figure 8D,E were attributed to UV–Vis and fluorescence spectra of the OX_2_-OH solutions in different pHs, respectively. Similar results of OX_1_-OH were observed for OX_2_-OH by decreasing the absorbance peak in 330–500 nm and increasing the absorbance peak in 400–600 nm by rising the pH from acidic (1–6) to neutral (7) and alkaline (8–14) values. In this regard, maximum intensity of absorbance peak in 330–500 nm was observed for the pH 1–5, and also the maximum intensity was recorded for pH 10–14 with absorbance peak in 400–600 nm, in addition to bathochromic or red shift phenomenon in *λ*_*max*_ (440 to 540 nm). The fluorescence spectra presented in Fig. 8E are in agreement with UV–Vis spectra, where the fluorescence intensity was increased as a result of increasing pH from acidic to alkaline conditions. The maximum emission was observed for OX_2_-OH in the media with pH of 12, 13, and 14. Figure 8F showed the photograph of colorimetric and fluorometric changes for OX_2_-OH solutions with pH of 1–14 under visible light and UV irradiation (365 nm). The intense coloration and fluorescence emission in alkaline than the acidic media was resulted from deprotonation and induction of negative charges to phenolate hydroxyl group in the chemical structure of OX_2_-OH (or OX_1_-OH), which induced higher conjugation and electron resonance in alkaline samples by increasing the absorbance intensity, bathochromism (red shift), and also higher fluorescence intensity.

**Figure 9:**
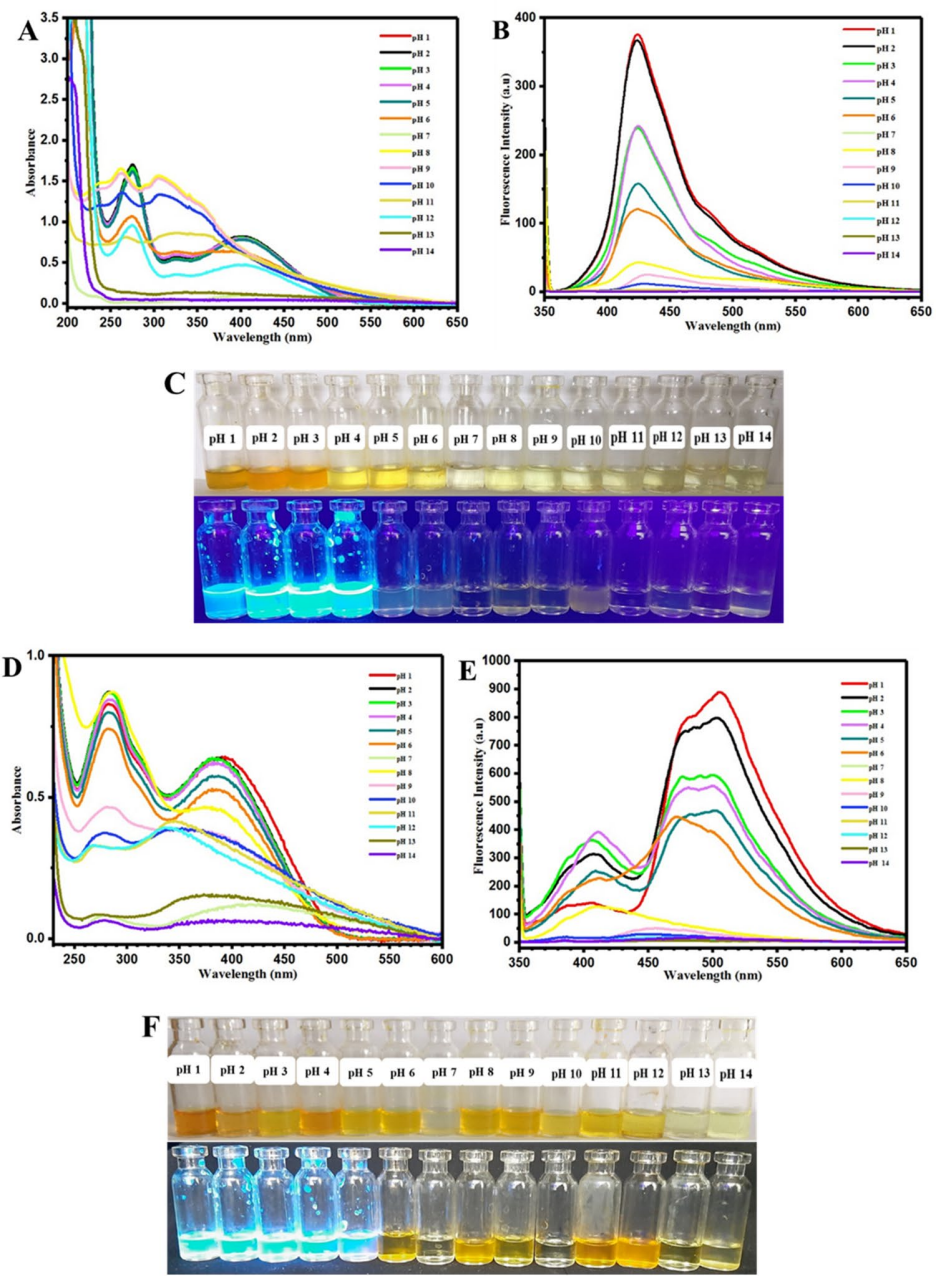
Photodetection of pH using UV–Vis and fluorescence spectroscopies for (**A**) and (**B**) OX_1_-Naph and (**D**) and (**E**) OX_2_-Naph solutions and also photographic images of colorimetric and fluorometric properties of (**C**) OX_1_-Naph and (**F**) OX_2_-Naph at pH of 1–14.

It is believed that chemical structure of oxazolidine, its substituted groups, and length of the conjugated structure can influence its optical properties and consequently its application in chemosensors. For this reason, the colorimetric and fluorometric characteristics of OX_1_-Naph and OX_2_-Naph were studied in aqueous solution with pH of 1–14, similar to OX_1_-OH and OX_2_-OH. The results for OX_1_-Naph and OX_2_-Naph are very different in comparison with the results for OX_1_-OH and OX_2_-OH. Increase of pH from acidic to alkaline pH was led to hypsochromism phenomena (blue shift) and also remarkable decrease of absorbance intensity in the case of alkaline solutions with higher pH values of 12, 13, and 14 (Fig. 9A,D). However, investigation of fluorescence emission for these chromophores indicated remarkable decrease of emission intensity by increasing pH from acidic to alkaline, and the minimum emission was obtained for the highly alkaline solutions with pH of more than 8 (Fig. 9B,E). The photograph of these solutions under visible light and UV irradiation (365 nm) are presented in Fig. 9C,F for OX_1_-Naph and OX_2_-Naph, respectively. The coloration and fluorescence emission for both of the OX_1_-Naph and OX_2_-Naph are in agreement with the results of UV–Vis and fluorescence spectra, where decrease of color and emission intensity was observed due to the increase of pH from acidic to alkaline. For example, both of the OX_1_-Naph and OX_2_-Naph solutions have highly intense yellow color under visible light and intense cyan blue fluorescence emission at pH of 1–5, which discoloration and fluorescence-off can be observed at pH of 6–14. These results can confirm potential applications of OX_1_-Naph and OX_2_-Naph in development of novel colorimetric and fluorometric chemosensors for photodetection of pH in aqueous media.

### Acidochromic security papers for anticounterfeiting technology

**Figure 10:**
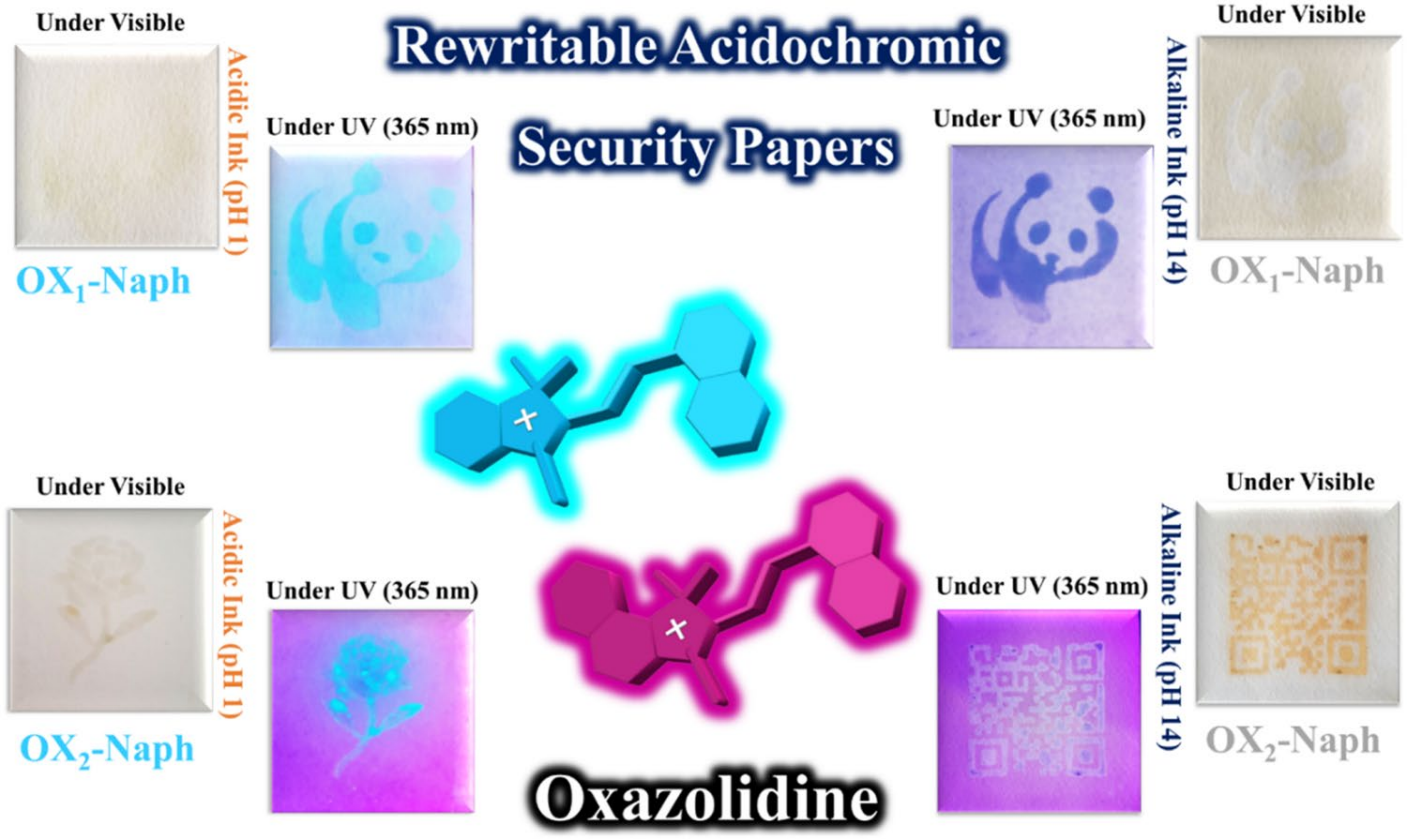
Printing of security marks and tags on OX_1_-Naph and OX_2_-Naph acidochromic papers by stamping of acidic (pH 1) and alkaline (pH 14) solutions as water-based inks.

According to the recent studies in the field of anticounterfeiting materials, oxazolidine derivatives have potential applications for development of hydrochromic security papers, acidochromic inks, and also fluorescent polymer nanoparticles as water-based anticounterfeiting inks^25,33,36,37^. Hence, the acidochromic papers based on OX_1_-OH, OX_2_-OH, OX_1_-Naph, and OX_2_-Naph were prepared by layer-by-layer coating of their solutions on the fluorescent-off cellulosic papers. According to the colorimetric and fluorometric properties of the oxazolidine derivatives that observed in the previous sections, these molecules displayed color change and fluorescence emission in response to pH variation. Therefore, the security marks and tags were printed on the acidochromic papers by stamping of acidic and alkaline solutions as water-based inks. As shown in Fig. 10, the acidochromic papers displayed both of the chromism and fluorescence emission after treatment by acidic and alkaline solutions. The printed security marks and tags on acidochromic papers coated with OX_1_-Naph showed highly cyan blue and highly intense dark blue fluorescence emission under UV irradiation by using of acidic and alkaline inks, respectively. In the case of acidochromic papers coated with OX_2_-Naph, highly intense cyan blue and white fluorescence emission were observed after printing of security marks by using acidic and alkaline inks, respectively. These papers have rewritable acidochromic characteristics, which discoloration was observed after evaporation of the water-based inks and the erased papers can be used again for printing of security marks.

**Figure 11:**
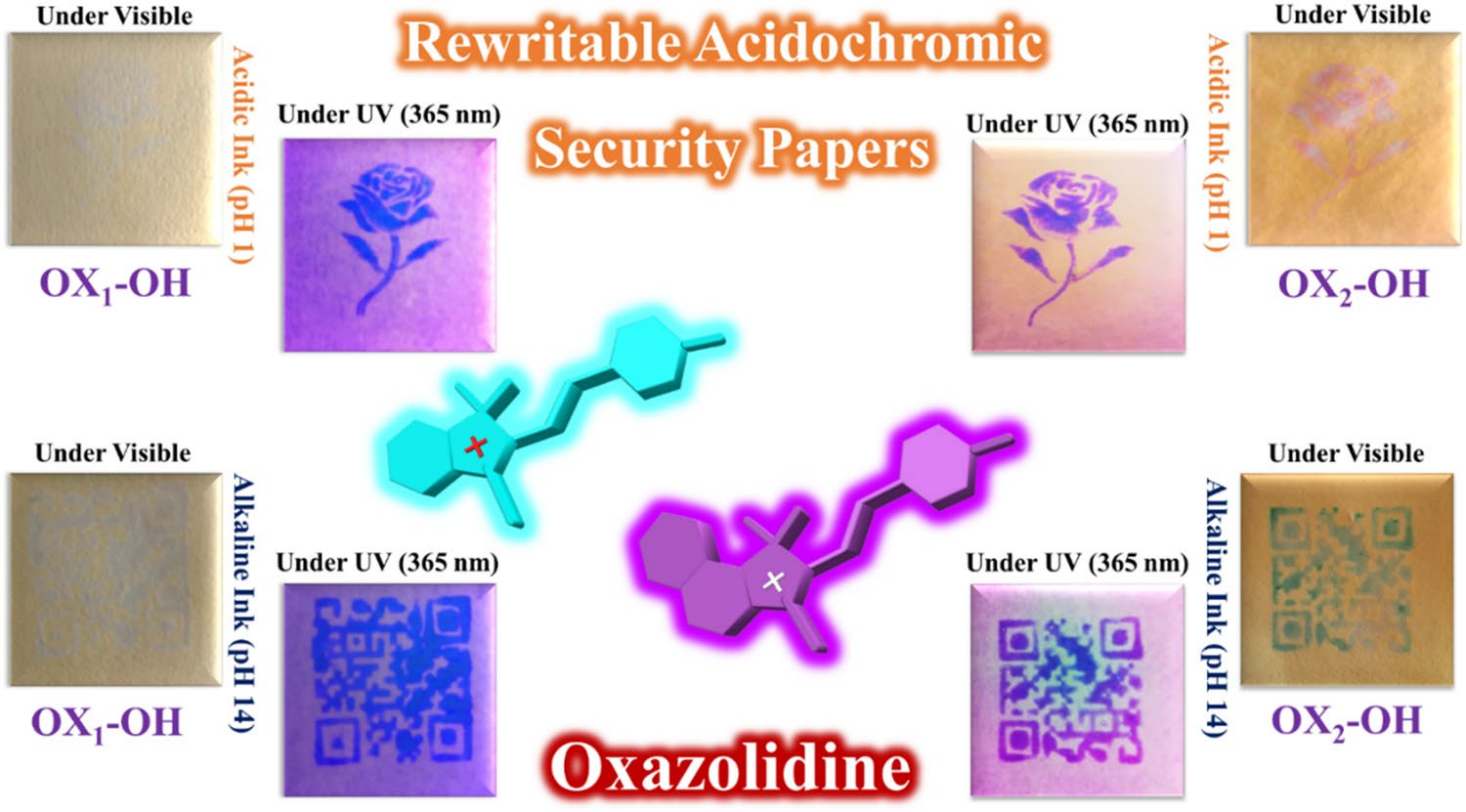
Printing of security marks and tags on OX_1_-OH and OX_2_-OH acidochromic papers by stamping of acidic (pH 1) and alkaline (pH 14) solutions as water-based inks.

Rewritable acidochromic papers based on OX_1_-OH and OX_2_-OH were used for printing of acidic and alkaline water-based inks by stamping, and the results are shown in Fig. 11. As expected, both of the acidochromic papers based on OX_1_-OH and OX_2_-OH displayed color change and fluorescence emission in response to acid and base treatment, where the printed security marks showed blue and purple fluorescence emission under UV irradiation, respectively. The sensitivity and intensity of rewritable hydrochromic papers based on OX_1_-OH and OX_2_-OH are more than the papers based on OX_1_-Naph and OX_2_-Naph. It can be attributed to major role of the protonation and deprotonation of hydroxyl functional groups after impregnation of paper with acid or base solutions. In fact, acid or base treatment act as electron donating and electron withdrawing groups in the case of hydroxyl-functionalized oxazolidine molecules that was observed as highly-intense color change and fluorescence emission^44–47^.

## Conclusion

Four oxazolidine derivatives (OX_1_-OH, OX_2_-OH, OX_1_-Naph, and OX_2_-Nph) were successfully synthesized, and their chemical structures were characterized by 1H NMR analysis. Investigation of solvatochromism and acidochromism phenomena for the oxazolidine molecules in different solvents (protic and aprotic) and aqueous solutions with various pH values (1–14) showed higher sensitivity of colorimetric and fluorometric properties to the polarity and pH of surrounding media. These molecules have potential applications in development of chemosensors for photodetection of polarity and pH of media by UV–Vis and fluorescence spectroscopies. Efficient physical interactions (van der Waals and hydrogen bonding) between oxazolidine and solvent molecules and also protonation and deprotonation of the substituted groups of oxazolidine molecules in acidic and alkaline media, respectively, were the main effective parameters. In addition, the acidochromic characteristics of the oxazolidine derivatives were used for development of rewritable acidochromic papers with potential applications in anticounterfeiting technology by using acid and base inks. For this purpose, the oxazolidine molecules were coated on the paper surface by layer-by-layer method, and the relevant security papers were utilized for printing of security tags by using of acidic and alkaline aqueous solutions as the inks. The printed marks and tags displayed coloration and fluorescence emission under visible and UV (365 nm) light, which are erasable after evaporation of the inks. The acidochromic papers were fully rewritable and can be also used for photodetection of pH in aqueous media as a paper-based chemosensor.
